# EF24, a Curcumin Analog, Reverses Interleukin-18-Induced miR-30a or miR-342-Dependent TRAF3IP2 Expression, RECK Suppression, and the Proinflammatory Phenotype of Human Aortic Smooth Muscle Cells

**DOI:** 10.3390/cells13201673

**Published:** 2024-10-10

**Authors:** Yusuke Higashi, Ryan Dashek, Patrice Delafontaine, Randy Scott Rector, Bysani Chandrasekar

**Affiliations:** 1 Medicine/Cardiology, Tulane University School of Medicine, New Orleans, LA 70112, USA; pdelafon@tulane.edu; 2 NextGen Precision Health, University of Missouri, Columbia, MO 65211, USA; dashekr@health.missouri.edu (R.D.); rectors@health.missouri.edu (R.S.R.); 3 Comparative Medicine Program, University of Missouri, Columbia, MO 65211, USA; 4 Research Service, Harry S. Truman Memorial Veterans Hospital, Columbia, MO 65201, USA; 5 Department of Medicine, Division of Gastroenterology and Hepatology, University of Missouri, Columbia, MO 65201, USA; 6 Department of Nutrition and Exercise Physiology, University of Missouri, Columbia, MO 65201, USA; 7 Department of Medicine, Division of Cardiovascular Medicine, University of Missouri School of Medicine, Columbia, MO 65201, USA; 8 Department of Medical Pharmacology and Physiology, University of Missouri, Columbia, MO 65201, USA; 9 Dalton Cardiovascular Center, University of Missouri, Columbia, MO 65203, USA

**Keywords:** curcumin, inflammation, cell proliferation, migration, vascular proliferative diseases, atherosclerosis

## Abstract

Curcumin, a polyphenolic compound derived from the widely used spice *Curcuma longa,* has shown anti-atherosclerotic effects in animal models and cultured vascular cells. Inflammation is a major contributor to atherosclerosis development and progression. We previously reported that the induction of the proinflammatory molecule TRAF3IP2 (TRAF3 Interacting Protein 2) or inhibition of the matrix metallopeptidase (MMP) regulator RECK (REversion Inducing Cysteine Rich Protein with Kazal Motifs) contributes to pro-oxidant, proinflammatory, pro-mitogenic and pro-migratory effects in response to external stimuli in vascular smooth muscle cells. Here we hypothesized that EF24, a curcumin analog with a better bioavailability and bioactivity profile, reverses interleukin (IL)-18-induced TRAF3IP2 induction, RECK suppression and the proinflammatory phenotype of primary human aortic smooth muscle cells (ASMC). The exposure of ASMC to functionally active recombinant human IL-18 (10 ng/mL) upregulated TRAF3IP2 mRNA and protein expression, but markedly suppressed RECK in a time-dependent manner. Further investigations revealed that IL-18 inhibited both miR-30a and miR-342 in a p38 MAPK- and JNK-dependent manner, and while miR-30a mimic blunted IL-18-induced TRAF3IP2 expression, miR-342 mimic restored RECK expression. Further, IL-18 induced ASMC migration, proliferation and proinflammatory phenotype switching, and these effects were attenuated by TRAF3IP2 silencing, and the forced expression of RECK or EF24. Together, these results suggest that the curcumin analog EF24, either alone or as an adjunctive therapy, has the potential to delay the development and progression of atherosclerosis and other vascular inflammatory and proliferative diseases by differentially regulating TRAF3IP2 and RECK expression in ASMC.

## 1. Introduction

Atherosclerotic vascular diseases, such as coronary artery disease and stroke, are significant health concerns globally due to their high prevalence and severe health consequences [1]. These diseases result from the buildup of plaque within arteries, impeding blood flow to vital organs like the heart and brain [2]. The impact of these diseases extends beyond individual’s health, straining healthcare systems and economies through increased healthcare costs, lost productivity, and premature deaths. Due to aging populations and a rise in risk factors associated with modern lifestyle, atherosclerotic vascular diseases continue to be a major public health challenge worldwide, emphasizing the importance of preventive measures and effective therapeutic strategies. However, current medical treatments are restricted to lifestyle changes, lipid-lowering therapy, or the management of risk factors such as hypertension and diabetes [3,4,5,6]. Therefore, the identification of potential therapeutic targets based on a deeper understanding of disease mechanisms is greatly needed.

Smooth muscle cells (SMCs) play a crucial role in the development of atherosclerosis, a condition characterized by plaque buildup within arterial walls [7,8]. Initially, atherosclerosis begins with ectopic lipid accumulation within the tunica intima (fatty streak) followed by the recruitment of pro-inflammatory cells, leading ultimately to vascular inflammation and arterial wall injury. In response to arterial wall injury, SMCs within intima and/or media layers migrate to the site of injury, proliferate, and contribute to intimal layer thickening and plaque development. Initially, the proliferating SMCs contribute to the formation of the fibrous cap covering the atherosclerotic plaque, thereby providing structural stability. As the plaque advances, SMCs secrete matrix-degrading metallopeptidases (matrix metalloproteinases or MMPs) and contribute to the degradation of tissue matrices within the plaque, potentially leading to plaque rupture and catastrophic consequences such as heart attack or stroke.

Interleukin-18 (IL-18) is a proinflammatory cytokine with pleiotropic effects. Its elevated systemic levels have been shown to serve as a risk factor for the development of cardiovascular diseases [9,10]. In combination with IL-12, IL-18 promotes a Th1 response [11] and elevates the production of pro-inflammatory mediators, including cytokines, chemokines, and adhesion molecules [12,13], resulting in the amplification of inflammatory responses. Accumulating evidence indicates an integral role for IL-18 in the development [14,15] and progression of atherosclerotic lesions, leading to the destabilization of plaques [16]. Though macrophages within a plaque are shown to express IL-18, its receptor, IL-18 receptor (IL-18R), is expressed by all plaque constituent cells, including SMCs [17], indicating that IL-18 could promote inflammatory responses in all cell types within a plaque. Therefore, understanding the role of IL-18 and its mechanisms of action may offer novel insights into therapeutic strategies for atherosclerosis development and subsequent clinical complications caused by a plaque rupture.

In light of IL-18-initiated inflammatory responses and tissue remodeling, we focused on understanding the roles and effects of targeting TRAF3IP2 (TRAF3 Interacting Protein 2) and RECK (Reversion Inducing Cysteine Rich Protein with Kazal Motifs) in aortic SMCs (ASMC). TRAF3IP2 is a cytoplasmic adapter protein and an upstream regulator of IKK/NF-κB and JNK/AP-1 [18,19]. We previously reported that TRAF3IP2 is a critical intermediate in IL-18-induced cardiac fibroblast migration and differentiation, mediated in part by the redox-sensitive NF-κB and AP-1-dependent signaling [20]. RECK is a membrane-anchored glycoprotein and an MMP regulator [21,22] known to inhibit the activation of various MMPs, including MMPs 2, 7, 9, and 14 (MT1-MMP) [21,22]. In cardiac fibroblasts, we previously showed that IL-18 downregulates RECK while upregulating MMP9 expression and activity, and preexposure to acetylsalicylic acid restored RECK expression and attenuated migration [23]. Interestingly, TRAF3IP2 and RECK are both expressed in ASMCs [24,25]; however, their roles in IL-18-induced ASMC migration, proliferation and proinflammatory phenotype modulation have not been fully investigated.

Curcumin, a polyphenolic compound derived from the widely used spice turmeric (*Curcuma longa*) has long been reported to exert antioxidant and anti-inflammatory effects in vivo and in vitro [26] with favorable cardiovascular beneficial effects [27,28,29,30]. However, the low bioavailability and bioactivity profile in vivo hampers its use as a preventative/therapeutic agent [31,32]. Therefore, several monocarbonyl analogues of curcumin have been developed. EF24 ((3E,5E)-3,5-bis[(2-fluorophenyl)methylidene]piperidin-4-one) is a synthetic monocarbonyl analogue of curcumin that caused improved bioavailability and bioactivity profile [32]. Its therapeutic potential has been tested in various preclinical models of cancer [33,34,35,36]. It also exerted proapoptotic, anti-inflammatory and anti-proliferative effects in in vitro studies using various cancer cell types. In a recent study, Zhang et al. tested the efficacy of 12 compounds, including the three curcumin analogs EF24, 2HBA, and HO-3867 on cellular senescence, and identified EF24 to be highly effective in blocking alveolar epithelial cell senescence [37], suggesting that EF24 exerts pleiotropic effects, and its effects are cell type-specific. Because SMC proliferation, migration and proinflammatory phenotype switching contribute to atherosclerosis development and progression, we hypothesized that EF24 could exert anti-atherosclerosis effects by inhibiting IL-18-induced ASMC proliferation, migration and proinflammatory phenotype switching, and determined the underlying molecular mechanisms.

## 2. Materials and Methods

### 2.1. Materials

EF24 was purchased from Sigma-Aldrich (#E8409) (St. Louis, MO, USA) and used at a final concentration between 1 and 10 μM for 12 h prior to the addition of interleukin (IL)-18. Reid and colleagues previously reported that the bioavailability of EF24 was about 60 and 35% after oral and intraperitoneal administration of EF24 in a mouse model [38]. In that study, pharmacokinetics revealed that a 10 mg/kg dose by oral, intravenous or intraperitoneal administration resulted in peak plasma levels of 2.5 μM. Fresh DMSO was used as a solvent to dissolve EF24. A stock solution of 10 mM in 100% fresh DMSO was prepared. In studies involving dose–response experiments with EF24, the final DMSO concentration was 0.1% when the highest concentration of EF24 used (10 μM). Since the dose–response studies revealed that 2.5 μM is optimal, in subsequent experiments, EF24 was used at a final concentration of 2.5 μM. In those experiments, the final DMSO concentration in controls was 0.025%. Since DMSO at 0.1% did not affect any of the parameters examined, we did not perform experiments using various concentrations of DMSA as controls. CELLDETH-RO Roche Cell Death Detection ELISA^PLUS^ kit (#11774425001) and all other biochemicals were purchased from Sigma-Aldrich. Carrier-free functionally active human recombinant human IL-18 (rhIL-18; #B001-5) was purchased from R & D Systems (Minneapolis, MN, USA) and used at a concentration of 10 ng/mL as previously described [39]. Normal goat IgG (#AB-108-C), goat anti-human IL-18Rα/IL-1R5 neutralizing antibody (#AF840), recombinant human IL-18BPa:fragment crystallizable region (Fc) chimera (119-BP-100) and recombinant human IgG1 Fc protein (#100-HG) were all purchased from R & D Systems and used at 10 μg/mL for 1 h prior to IL-18 addition [39,40]. The p38 MAPK inhibitor SB239063 (#S7741; 10 μM in fresh DMSO for 1 h), the JNK inhibitor SP600125 (#S1460, 20 μM in fresh DMSO for 30 min), and the ERK inhibitor PD98059 (#S11277; 10 μM in fresh DMSO for 1 h) were purchased from Selleckchem.com (Houston, TX, USA). BioCoat^™^ Matrigel^™^ invasion chambers (#354481) were from BD/Discovery Labware (Bedford, MA, USA). The Gibco™ ITS-G (Insulin-Transferrin-Selenium, #41400045) supplement, Pierce™ BCA Protein Assay Kit (#23227), Trypan blue solution, 0.4% (#15250061), Invitrogen™ Human Caspase-3 (cleaved) ELISA Kit (# KHO1091), and Invitrogen™ CyQUANT™ LDH Cytotoxicity Assay, fluorescence (#C20302), CyQUANT™ Cell Proliferation Assay (#C7026), MagMax™ *mir*Vana™ miRNA Isolation Kit (#A27828), SuperSignal^®^ West Femto Maximum Sensitivity Substrate (#34096), and protein molecular weight markers were all purchased from Thermo Fisher Scientific (Waltham, MA, USA).

### 2.2. Cell Culture

Human primary aortic SMC (ASMC) was purchased from LONZA (#CC-2571, Basel, Switzerland) and cultured in SmGM™-2 Smooth Muscle Cell Growth Medium-2 with SingleQuots™ supplements containing growth factors (#CC-4149) as described previously [25,41]. Real-time quantitative PCR (RT-qPCR) was performed using inventoried Applied Biosystems™ TaqMan^®^ probes (Thermo Fisher Scientific), and revealed that ASMCs were positive for α smooth muscle actin (αSMA) and smooth muscle myosin heavy chain (SM-MHC), but negative for Von Willebrand Factor, an endothelial marker. At 70–80% confluency, cells were made quiescent by incubating in basal medium containing 0.5% BSA or ITS-G (Gibco™ Insulin-Transferrin-Selenium) supplement at 1× for 48 h, and then exposed to IL-18 (10 ng/mL) or IL-18+EF24. ITS-G (Gibco™ Insulin-Transferrin-Selenium), instead of 0.5% BSA, was employed to induce quiescence in experiments involving MMPs, because albumin has been reported to induce MMP9 and TIMPs in various cell types [42,43,44,45].

### 2.3. Adenoviral Vectors

Adenovirus-expressing shRNA that targets human TRAF3IP2 (Ad-GFP-U6-h-TRAF3IP2-shRNA or Ad.TRAF3IP2-shRNA) was custom-generated at Vector Biolabs (Malvern, PA, USA). The control Ad.GFP (#1060) was purchased from Vector Biolabs. Adenoviral vector-expressing human RECK cDNA (GenBank accession # NM_021111) was also custom-generated at Vector Biolabs. Ad.GFP shRNA and Ad.eGFP served as respective controls. To target TRAF3IP2 or overexpress RECK, ASMCs were transduced with respective adenoviruses at moi10 for 48 or 24 h, respectively. The knockdown of TRAF3IP2 and overexpression of RECK were confirmed by RT-qPCR and Western blotting. Adenoviral vectors expressing siRNA against MMP2 (Ad.siMMP2, 5′-AACGGACAAAGAGTTGGCAGTATCGATACTGCCAACTCTTTGTCCGTT-3′ [46]), MMP9 (Ad.siMMP9) and GFP (Ad.siGFP) have been previously described [47], and used at moi 100 for 48 h. At the indicated moi, the transduction with adenoviral vectors at the indicated moi did not affect cell shape, viability, or attachment to the culture dish (Supplementary Figure S1).

### 2.4. mRNA Expression

DNA-free total RNA was isolated using the RNeasy Plus Micro Kit (#74034; Qiagen, Germantown, MD, USA). Agilent 2100 Bioanalyzer (Agilent Technologies, Palo Alto, CA, USA) was used to analyze the quality of RNA. Two micrograms of total RNA with an RNA integrity number of 9.0 or greater (scale = 1–10) were reverse-transcribed into single-stranded cDNA and used in RT-qPCR employing Applied Biosystems™ TaqMan^®^ probes—*TRAF3IP2* (Assay ID: Hs00974570_m1), *RECK* (Hs01019185), *ACTA2* (Hs00426825_g1), *MYH11* (Hs00975796_m1), Galectin 3 (*LGALS3*; Hs03680062_m1), *VCAM1* (Hs00164932_m1), *OLR1* (Hs01552593_m1), *CCL2* (MCP1; Hs07292220_s1), *Il6* (Hs00174131_m1), *Il8* (CXCL8; Hs00174103_m1), and *TNF-α* (Hs00174128_m1). Both *GAPDH* (Hs02786624_g1) and *ACTB* (β-actin; Hs01060665_g1) served as housekeeping genes. Samples were run in triplicate, and samples without RNA and samples processed without RT served as negative controls. Data were analyzed using the delta–delta CT (2^−ΔΔCt^) method, normalized to corresponding *GAPDH* or β-actin expression and presented as fold change from untreated control.

### 2.5. Micro RNA Expression, Mimics, and Transfections

For miRNA expression analysis, total RNA enriched with miRNA was isolated using the MagMax™ *mir*Vana™ miRNA Isolation Kit. miR-30a and miR-342 expression levels were analyzed by RT-qPCR using Applied Biosystems™ TaqMan^®^ probes—miR-30a (assay ID: Hs04231430_sH) and miR-342 (Hs04231562_s1). Expression levels of U6 (Assay ID: 001973) served as a loading control. Data were normalized to corresponding U6 expression and presented as fold change from untreated control. The miR30a mimic (Assay ID: MH11062, mature miRNA sequence: UGUAAACAUCCUCGACUGGAAG), the miR-342 mimic (Assay ID: MC13066, mature miRNA sequence: AGGGGUGCUAUCUGUGAUUGA) and miRNA mimic negative control (NC, Invitrogen™ *mir*Vana™ miRNA Mimic, Negative Control #1; #4464058) were all purchased from Applied Biosystems/Thermo Fisher Scientific. According to the manufacturer’s website, the negative control (NC) contains a random sequence miRNA mimic sequence that has been extensively tested to not affect known miRNA function in various human cell lines and tissues. ASMCs were transfected with 80 nM of mimics or NC using the Neon^®^ Transfection System (MPK-5000; Invitrogen/Thermo Fisher Scientific, Waltham, MA, USA) using the following parameters: pulse voltage—1300 V; pulse width—20 ms; pulse number—2; the tip type—10 μL. They were then cultured for 24 h. The transfection efficiency of ASMC was ∼59% with 2% cell death, as determined using the pEGFP-N1 vector (#6081-5; Addgene, Watertown, MA, USA). Transfections with the indicated mimics or the NC had no significant effects on ASMC shape, viability, or adherence (trypan blue dye exclusion, Supplementary Figure S1).

### 2.6. Western Blot Analysis and Quantification of Cleaved Caspase-3 Levels

Western blotting using 20 μg of cleared whole-cell lysates was performed as previously described [20,22,23,24,25,39,40,41,47]. The following antibodies were used: TRAF3IP2 (1:400; NB100–56740, Novus Biologicals, LLC, Centennial, CO, USA), Tubulin (1:1000; #2144, Cell Signaling Technology/CST, Danvers, MA, USA), RECK (1 : 1000; #3433, CST), MMP2 (1 : 500; #ab97779, Abcam, Waltham, MA, USA), MMP9 (1 : 1000; #2270, CST), and GAPDH (1:1000; #NB300-221, Novus Biologicals, LLC). The intensity of immunoreactive bands was semiquantified using densitometry, and the densitometric data from an indicated number of independent experiments were summarized and presented as a ratio and fold change of a specific protein to a corresponding internal control. Approximate molecular weights of target proteins are indicated on the right. Cleaved caspase-3 levels were quantified in cleared whole-cell lysates according to the manufacturer’s instructions using the Caspase 3 (Cleaved) Human ELISA Kit. The analytical sensitivity of this sandwich ELISA kit was 0.033 ng/mL, with an assay range of 0.039–2.5 ng/mL.

### 2.7. Cell Proliferation, Migration and Phenotypic Modulation

ASMC proliferation was analyzed by the CyQUANT™ Cell Proliferation Assay [25]. Briefly, ASMCs were seeded at 1 × 10^3^ cells/well in 200 μL of complete medium in 96-well flat-bottom plates with a clear bottom and black sides (VWR Scientific Products, West Chester, PA, USA), and incubated for 24 h to allow attachment. The complete medium was then replaced with medium containing 0.5% BSA and incubated for an additional 48 h to induce quiescence. The quiescent cells were then incubated with IL-18 at 10 ng/mL for 48 h. The media were then removed, and the plates frozen for 2 h at −80 °C prior to assay. The plates were then thawed, stained with CyQUANT™ GR dye according to the manufacturer’s protocol, and read at 485/20 excitation and 528/20 emission in a microplate fluorescence reader (Bio-Tek Instruments, Winooski, VT, USA). The data were analyzed using the KC^4^ 3.4 software (Bio-Tek Instruments, Winooski, VT, USA). In a subset of experiments, ASMCs were transduced with Ad.TRAF3IP2 shRNA or Ad.GFP shRNA (moi10 for 48 h) prior to IL-18 addition at 10 ng/mL for 48 h. To determine if the ectopic expression of RECK inhibits IL-18-induced ASMC proliferation, ASMCs were transduced with Ad.RECK or Ad.eGFP at moi 10 for 24 h, made quiescent, and then treated with IL-18. To determine if EF24 inhibits IL-18-induced ASMC proliferation, quiescent ASMCs were exposed to EF24 at 2.5 μg/mL in DMSO for 1 h, followed by the addition of IL-18 at 10 ng/mL for 48 h.

### 2.8. Cell Migration

To determine the effects of IL-18 on ASMC migration, we employed BioCoat™ Matrigel™ invasion chambers (Corning Inc., Massachusetts, MA, USA) with 8.0 μm pore polyethylene terephthalate membranes and a thin layer of Matrigel™ basement membrane matrix, as described previously [24,39]. In brief, ASMCs were trypsinized, suspended in a basal medium containing 0.5% BSA, and layered (2.0 × 10^5^ cells/mL) on the coated insert filters. The cells were then exposed to IL-18 (10 ng/mL). The lower chamber contained 20% serum. After incubation for 18 h at 37 °C, the membranes were washed in PBS, and non-invading cells on the upper surface were scraped off using cotton swabs and stained with hematoxylin. ASMCs migrating to the lower surface of the membrane were counted in 10 independent fields at 20× magnification and summarized as mean ± SEM.

In experiments where the role of TRAF3IP2 is determined, ASMCs were transduced with Ad.TRAF3IP2 shRNA or Ad.GFP shRNA at moi 10 for 48 h, made quiescent, layered on Matrigel™ basement membrane, and then treated for 18 h with IL-18 (10 ng/mL). To determine whether the ectopic expression of RECK blunts IL-18-induced ASMC migration, ASMCs were transduced with Ad.RECK or Ad.eGFP at moi 10 for 24 h, made quiescent, layered on Matrigel basement membrane, and then treated with IL-18 at 10 ng/mL for 18 h. To determine if EF24 inhibits IL-18-induced ASMC migration, quiescent ASMCs layered on Matrigel basement membrane were treated with EF24 at 2.5 μM in DMSO for 1 h and then exposed to IL-18 at 10 ng/mL for 18 h. To investigate the role of MMPs 2 and 9 in IL-18-induced ASMC migration, ASMCs were transduced with Ad.siMMP2 or Ad.siMMP9 or the control Ad.siGFP at moi 100 for 48 h, made quiescent, and then used in the assay.

### 2.9. Phenotypic Modulation

To determine if IL-18 induces proinflammatory phenotype modulation and whether this effect is attenuated by TRAF3IP2 knockdown or the ectopic overexpression of RECK or treatment with EF24, low-passage ASMCs were infected with Ad.TRAF3IP2 shRNA (moi10 for 48 h) or Ad.RECK (moi10 for 24 h), or treated with EF24 at 2.5 μM in DMSO for 1 h prior to the addition of IL-18 at 10 ng/mL for 48 h. Ad.GFP-shRNA, Ad.GFP and DMSO served as respective controls. Total RNA was isolated and analyzed for the expression levels of SMC markers ACTA2 and MYH11, and the proinflammatory markers Galectin 3, OLR1, VCAM1, CCL2 (MCP-1), IL-6, IL-8, and TNF-α by RT-qPCR. The mRNA expressions data were normalized to corresponding β-actin expression and presented as fold change from corresponding untreated controls.

### 2.10. Cell Viability

After the indicated treatments, the effects of EF24 on cell viability were analyzed by 4 different methods. Firslty, trypan blue dye exclusion was used, whereby cells with intact membranes exclude the dye. The assay was performed using 0.4% trypan blue solution from Thermo Fisher Scientific. Cell viability was also determined by quantifying cleaved caspase-3 levels using a commercially available kit from Thermo Fisher Scientific. The assay was carried out as per the instructions provided by the manufacturer, and the levels were represented in pg/mL. Cell viability was also analyzed by quantifying mono- and oligonucleosomal fragmented DNA using a commercially available kit from Sigma-Aldrich. The assay was carried out as per the instructions provided by the manufacturer. LDH is a cytosolic enzyme that is released into the cell culture medium upon damage to the plasma membrane. LDH levels in media were determined using CyQUANT™ LDH Cytotoxicity Assay according to the manufacturer’s instruction. Results were normalized to cultures treated with 0.2% triton-X for 10 min and considered 100% of LDH release.

### 2.11. Statistical Analysis

All data were analyzed using the GraphPad Prism 8 software (San Diego, CA, USA) and presented as means ± SEM. Statistical significance was determined by a two-way ANOVA, followed by Fisher’s least significant difference test and Bonferroni’s post hoc analysis for multiple comparisons. Unpaired two-tailed Student’s *t*-test was performed for single comparisons. Both the normality of distribution and equality of variance were considered prior to performing statistical analysis. For example, in gene expression analyses via RT-qPCR, there were similar numbers of replicates. No samples were deleted. Differences are considered significant if the *p* value is <0.05.

## 3. Results

### 3.1. IL-18 Differentially Regulates TRAF3IP2 and RECK Expression in ASMC

**Figure 1:**
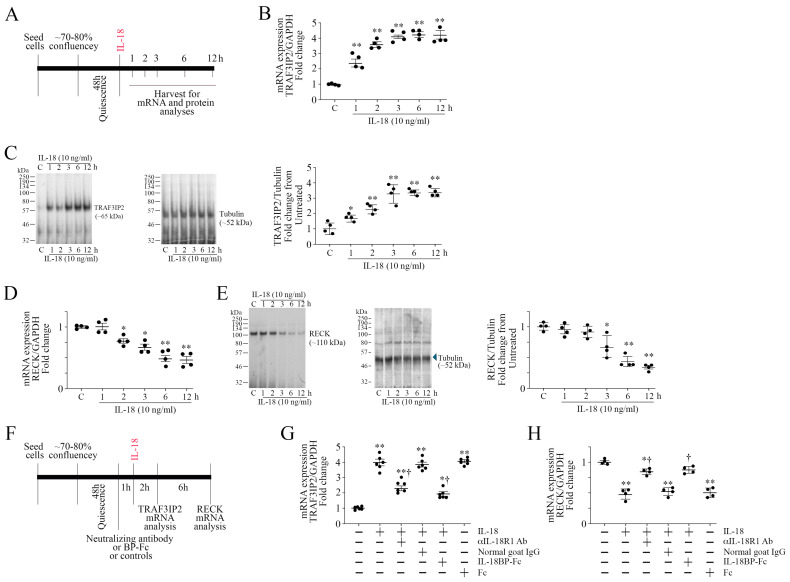
Interleukin-18 (IL-18) upregulates TRAF3IP2 but suppresses RECK expression in primary human aortic smooth muscle cells (ASMC). (**A**–**C**) IL-18 upregulates TRAF3IP2 expression. ASMCs were grown in complete media, and at 70–80% confluency, made quiescent for 48 h, and then incubated with rhIL-18 (10 ng/mL) for up to 12 h (experimental design in (**A**)). TRAF3IP2 mRNA expression was analyzed by RT-qPCR using a TaqMan™ probe (**B**) and its protein levels by Western blotting (**C**), with GAPDH and Tubulin serving as loading controls, respectively. * *p* < 0.05, ** at least *p* < 0.01 vs. untreated controls (*n* = 4). (**D**,**E**) Quiescent ASMCs were incubated with IL-18 as in (A) and were analyzed for RECK mRNA expression by RT-qPCR using a TaqMan™ probe (**D**) and protein levels by Western blotting (**E**). * *p* < 0.05, ** at least *p* < 0.01 vs. untreated controls (*n* = 4). (**F**–**H**) Specificity of IL-18 on TRAF3IP2 induction (**G**) and RECK suppression (**H**) was verified by incubating with neutralizing IL-18R1 antibody or IL-18BP-Fc chimera for 1 h prior to IL-18 addition for 2 (**G**) or 6 h (**H**), with normal goat IgG or Fc serving as controls. mRNA expressions of TRAF3IP2 and RECK were analyzed by RT-qPCR. (**C**,**E**) While a representative immunoblot is shown, the intensities of immunoreactive bands from 4 independent experiments were semiquantified by densitometry and are summarized on the right. (**G**) * *p* < 0.05, ** *p* < 0.01 vs. untreated controls, † *p* < 0.01 versus IL-18 (n = 6); (**H**) * *p* < 0.05, ** *p* < 0.01 vs. untreated controls, † *p* < 0.01 versus IL-18 (*n* = 4).

As shown in Figure 1A, ASMCs were made quiescent, exposed to the proinflammatory and proatherogenic cytokine IL-18 for up to 12 h, and then analyzed for TRAF3IP2 and RECK expressions by RT-qPCR and Western blotting. The results show that IL-18 induced TRAF3IP2 mRNA expression in a time-dependent manner (Figure 1B). A significant increase in its mRNA levels was detected at 1 h, which further increased at 2 h, and peaked at 3 h. No further increase was detected at 6 h, and the levels remained at these high levels for up to 12 h. Similarly, IL-18 upregulated TRAF3IP2 protein levels (Figure 1C), with peak levels detected around 3 h. In contrast to TRAF3IP2 expression, IL-18 suppressed RECK mRNA (Figure 1D) and protein expression (Figure 1E) in a time-dependent manner, with maximal suppression observed at 6 h. No further inhibition was seen at 12 h. In panels 1C and 1E, densitometric results from four independent experiments are summarized, as shown on the right. To determine if the effects were specific to IL-18, ASMCs were incubated with the ligand-binding IL-18R1 neutralizing antibodies or IL-18BP-Fc chimera prior to IL-18 addition (experimental design in Figure 1F). The results in Figure 1G,H show that IL-18R1 neutralizing antibodies and IL-18BP-Fc chimera each markedly reversed TRAF3IP2 induction and RECK suppression. Together, these data indicate that IL-18, while enhancing TRAF3IP2 expression, suppresses RECK in ASMCs (Figure 1).

### 3.2. TRAF3IP2 Knockdown or Ectopic Expression of RECK Reverses IL-18-Induced ASMC Proliferation and Migration

**Figure 2:**
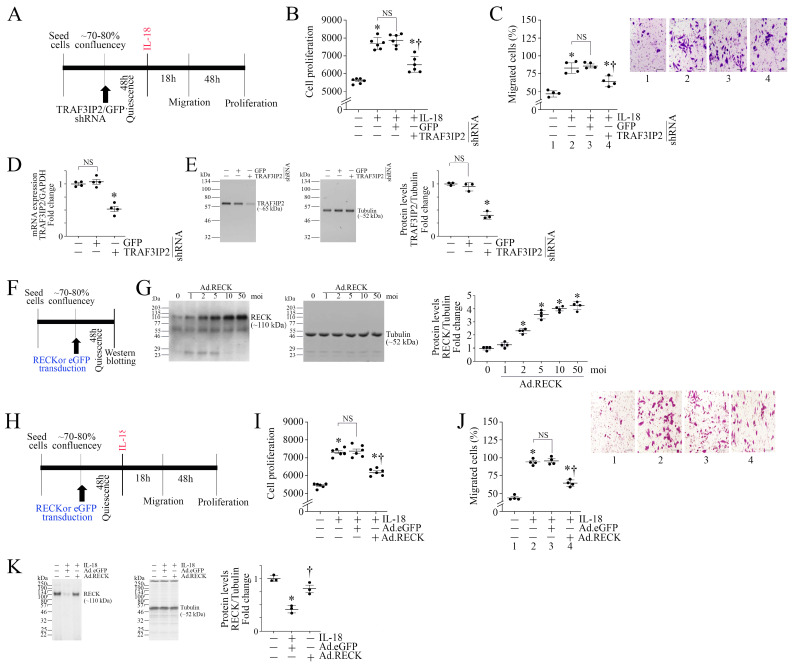
Targeting TRAF3IP2 or RECK overexpression blunts IL-18-induced ASMC proliferation and migration. (**A**–**E**) Silencing TRAF3IP2 inhibits IL-18-induced ASMC proliferation (**B**) and migration (**C**). ASMC were transduced with adenovirus-expressing shRNA targeting human TRAF3IP2 (moi 10 for 48 h), made quiescent, and then exposed to IL-18 (10 ng/mL; experimental design in (**A**)). ASMC proliferation was assessed after 48 h of IL-18 addition using the CyQUANT Cell proliferation assay (**B**), and migration after 18 h using Boyden chamber assay (**C**). ASMCs migrating to the lower surface of the membrane were counted in 10 different fields and summarized as mean ± SEM. (**B**,**C**) * *p* < at least 0.01 vs. Untreated; † *p* < 0.01 vs. IL-18 or IL-18+GFP (n = 6). Knockdown of TRAF3IP2 was confirmed by RT-qPCR using a TaqMan™ probe (**D**) and Western blotting (**E**). (**D**,**E**) * *p* < 0.01 vs. untreated (n = 3). (**F**,**G**) Dose-dependent effects of Ad.RECK on RECK expression (experimental design in (**F**)). Induction of RECK following adenoviral transduction was confirmed by Western blotting with tubulin serving as an internal control (**G**). (**H**–**K**) Forced expression of RECK inhibits IL-18-stimulated ASMC proliferation and migration. ASMCs were transduced with adenovirus-expressing human RECK cDNA (moi 10 for 24 h), made quiescent, and then treated with IL-18 (experimental design in (**H**)) and analyzed for proliferation (**I**) and migration (**J**) as in (**B**,**C**). (**C**,**J**) The insets show representative images of Matrigel™ Transwell invasion. Scale bar: 20 μM. (**E**,**G**,**K**) While a representative immunoblot is shown, the intensities of immunoreactive bands from three (**E**), four (**G**) and three (**K**) independent experiments were semiquantified by densitometry and are summarized on the right. (**I**) * *p* < at least 0.01 vs. Untreated; † *p* < 0.01 vs. IL-18 or IL-18+GFP (n = 6); (**J**) * *p* < at least 0.01 vs. Untreated; † *p* < 0.01 vs. IL-18 or IL-18+eGFP (n = 4); (**K**) * *p* < 0.01 vs. Untreated; † *p* < 0.05 vs. IL-18 or IL-18+eGFP (n = 3).

We have previously demonstrated that IL-18 exerts pro-mitogenic and pro-migratory effects in various cell types [20,23,39]. Figure 2A shows the experimental design, and the results demonstrate that IL-18 significantly induced ASMC proliferation (Figure 2B) and migration (Figure 2C, the inset shows representative images of Matrigel™ Transwell invasion assay), effects that were significantly attenuated by TRAF3IP2 knockdown. The knockdown of TRAF3IP2 was confirmed by RT-qPCR (Figure 2D) and Western blotting (Figure 2E; densitometric results from three independent experiments are summarized in the right-hand panel). Since IL-18 suppressed RECK expression (Figure 1D,E), we then investigated if RECK overexpression would reverse IL-18-induced ASMC proliferation and migration. At first, we determined the optimal moi needed for Ad.RECK transfection. Therefore, ASMCs were transduced with Ad.RECK or Ad.eGFP as shown in Figure 2F, and analyzed for RECK expression by Western blotting. Confirming our previous results [47], the forced expression of RECK by adenoviral transduction increased RECK expression in a dose-dependent manner, with peak levels of expression detected at moi10 (Figure 2G, densitometric results from three independent experiments are summarized in the right-hand panel). Therefore, in all subsequent experiments, Ad.RECK was used at moi 10. As shown in Figure 2H, Ad.RECK at moi 10 markedly reduced the promitogenic (Figure 2I) and pro-migratory effects (Figure 2J, the inset shows representative images of Matrigel™ Transwell invasion assay) of IL-18. Importantly, ectopically expressed RECK levels were not modulated by IL-18 (Figure 2K). Together, these results indicate that silencing TRAF3IP2 and ectopically expressed RECK each markedly attenuated IL-18-induced ASMC proliferation and migration (Figure 2).

### 3.3. Silencing TRAF3IP2 Reverses IL-18-Induced Suppression in SMC Markers and Inhibits the Induction of Proinflammatory Phenotype Markers

**Figure 3:**
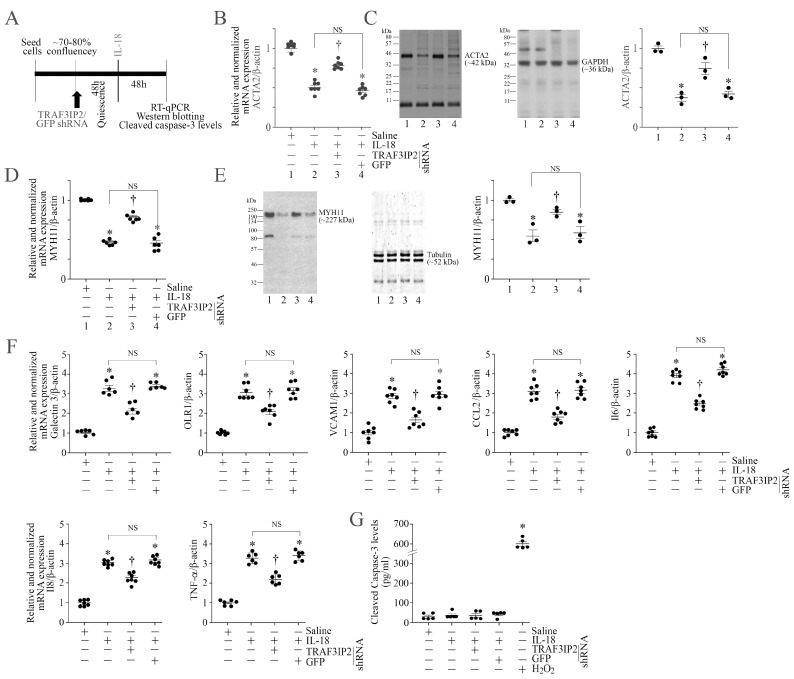
TRAF3IP2 knockdown restores SMC marker expression and inhibits ASMC proinflammatory phenotype without affecting cell viability. (**A**–**G**) Silencing TRAF3IP2 restores IL-18-mediated suppression in SMC markers, but inhibits the expression of proinflammatory phenotype markers, without significantly modulating cell viability. ASMCs were transduced with adenoviral TRAF3IP2 shRNA (moi10 for 48 h), made quiescent and then treated with IL-18 (10 ng/mL for 48 h; experimental design in (**A**)). Expressions of the SMC markers ACTA2 (**B**,**C**) and MYH11 (**D**,**E**) were analyzed by both RT-qPCR (**B**,**D**) and Western blotting (**C**,**E**). The proinflammatory phenotype markers Galectin 3, Olr1, VCAM, CCL2, IL-6, IL-8, and TNF-α were analyzed by RT-qPCR using TaqMan™ probes (**F**). Cell viability was assessed by analyzing cleaved caspase-3 levels using a commercially available Caspase-3 (Cleaved) Human ELISA (**G**). H_2_O_2_ (100 μM for 18 h) served as a positive control and induced a significant increase in cleaved capase-3 levels. (**C**,**E**) While a representative immunoblot is shown, the intensities of immunoreactive bands from three independent experiments were semiquantified by densitometry and are summarized on the right. (**B**,**D**,**F**,**G**) * *p* < at least 0.01 vs. Untreated; † *p* < 0.01 vs. IL-18 or IL-18+GFP (n = 6 or 7). (**C**,**E**) * *p* < 0.05 vs. Untreated; † *p* < 0.05 vs. IL-18 or IL-18+GFP (n = 3).

In an inflammatory and atherogenic environment, vascular SMCs undergo phenotype switching by losing their SMC marker expression and gaining the proinflammatory phenotype markers. Since TRAF3IP2 is a proinflammatory adapter molecule, we next investigated whether targeting TRAF3IP2 reverses IL-18-induced suppression in SMC markers and inhibits the induction of proinflammatory phenotype markers. While the experimental design is shown in Figure 3A, the results in Figure 3B–E show that exposure to IL-18 markedly suppressed the expression of SMC markers ACTA2 (Figure 3B,C) and MYH11 (Figure 3D,E) at both mRNA and protein levels, and that these inhibitory effects were reversed by TRAF3IP2 knockdown (Figure 3B–E). Further, treatment with IL-18 upregulated the mRNA expression of proinflammatory markers Galectin-3, OLR1, VCAM1, CCL2 (MCP-1), IL-6, IL-8, and TNF-α, and silencing TRAF3IP2 reversed these effects (Figure 3F) without affecting cell viability, as evidenced by the low cleaved caspase-3 levels (Figure 3G). However, H_2_O_2_, used as a positive control, significantly increased caspase-3 levels (Figure 3G). Together, these results indicate that TRAF3IP2 contributes to the IL-18-induced proinflammatory phenotype modulation of ASMC (Figure 3).

### 3.4. Ectopic Expression of RECK Reverses IL-18-Induced Suppression in SMC Markers and Inhibits the Induction of Proinflammatory Phenotype Markers

**Figure 4:**
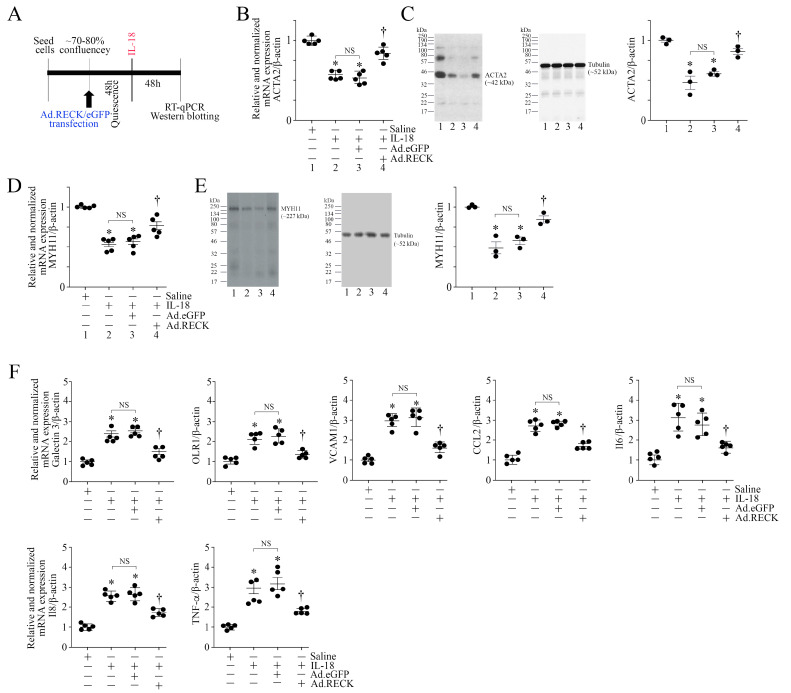
Ectopic expression of RECK blunts IL-18-induced inhibition in the expression of SMC markers and the induction of proinflammatory phenotype markers. (**A**–**F**) Forced expression of RECK restores IL-18-induced suppression of SMC markers and inhibits the induction of proinflammatory phenotype markers. ASMCs were transduced with adenovirus-expressing human RECK cDNA (moi10 for 24 h), made quiescent, and then treated with IL-18 at 10 ng/mL for 48 h (experimental design in (**A**)). The expression levels of SMC markers ACTA2 (**B**,**C**) and MYH11 (**D**,**E**) were analyzed by RT-qPCR (**B**,**D**) and Western blotting (**C**,**E**). The proinflammatory phenotype markers Galectin 3, Olr1, VCAM, CCL2, IL-6, IL-8, and TNF-α were analyzed by RT-qPCR using TaqMan™ probes (**F**). (**C**,**E**) While a representative immunoblot is shown, the intensities of immunoreactive bands from three independent experiments were semiquantified by densitometry and are summarized on the right. (**B**,**D**,**F**) * *p* < at least 0.01 vs. Untreated; † *p* < 0.01 vs. IL-18 or IL-18+eGFP (n = 5). (**C**,**E**) * *p* < 0.05 vs. Untreated; † *p* < 0.05 vs. IL-18 or IL-18+eGFP (n = 3).

RECK is not only an MMP regulator, but also an inhibitor of proinflammatory signaling. Since IL-18 inhibited RECK expression in ASMCs (Figure 1D,E), we next investigated whether the forced expression of RECK blunts IL-18 induced changes in ASMCs, including the proinflammatory phenotype markers. Indeed, the ectopic expression of RECK by an adenoviral vector (Figure 4A, experimental design) reversed IL-18-induced suppression in the expression of SMC markers ACTA2 (Figure 4B,C) and MYH11 (Figure 4D,E) at the mRNA and protein levels. Further, the forced expression of RECK reversed IL-18-induced upregulation in proinflammatory marker gene expression, which included Galectin-3, OLR1, VCAM1, CCL2, IL-6, IL-8, and TNF-α (Figure 4F). These results indicate that enhancing RECK expression restores IL-18-induced suppression in SMC markers and blunts the proinflammatory phenotype switching of ASMCs (Figure 4).

### 3.5. IL-18 Inhibits miR-30a and miR-342 Expression via Activation of Stress Activated Kinases

**Figure 5:**
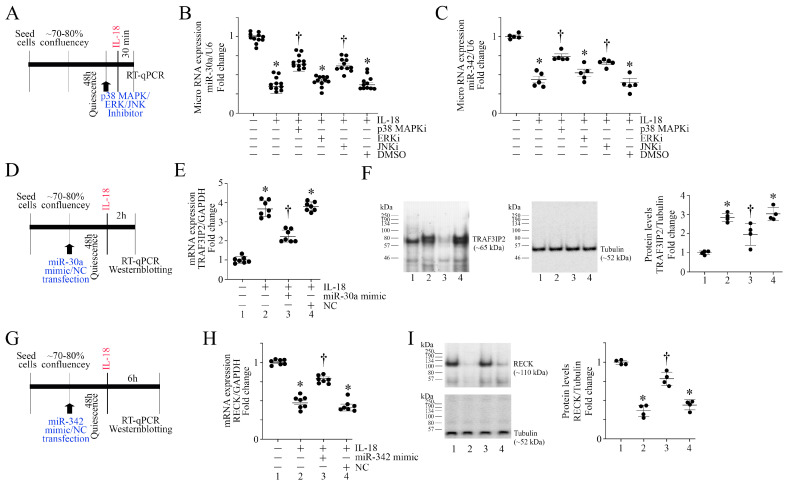
IL-18 inhibits miR-30a and miR-342 expression via stress-activated kinases. (**A**–**C**) IL-18 inhibits miR-30a and miR-342 expression via stress-activated kinases. Quiescent ASMCs were treated with inhibitors of either p38 MAPK (SB239063, 10 μM in DMSO for 1 h), ERK1/2 (SCH772984 10 μM in DMSO for 1 h) or JNK (SP600125, 20 μM in DMSO for 1 h) prior to IL-18 addition at 10 ng/mL for 30 min (experimental design in (**A**)). (**B**,**C**) Fresh DMSO (0.1%) served as a solvent control. miR-30a and miR-342 expressions were analyzed by TaqMan*®* Advanced miRNA assays, with U6 serving as a loading control. (**B**) * *p* < 0.001 vs. untreated, † *p* < at least 0.01 vs. IL-18 or IL-18+DMSO (n = 11), (**C**) * *p* < 0.01 vs. untreated, † *p* < at least 0.05 vs. IL-18 or IL-18+DMSO (n = 5). (**D**–**F**) miR-30a mimic inhibits IL-18-induced TRAF3IP2 expression. ASMC were transfected with miR-30a mimic (80 nM), made quiescent and then exposed to IL-18 at 10 ng/mL for 2 h (experimental design in (**D**)). TRAF3IP2 mRNA expression was analyzed by RT-qPCR (E) and its protein levels by Western blotting (**F**). (**G**–**I**) miR-342 mimic restores IL-18-induced RECK suppression. ASMCs were transfected with miR-342 mimic (80 nM), made quiescent and then exposed to IL-18 at 10 ng/mL for 6 h (experimental design in (**G**)). RECK mRNA expression was analyzed by RT-qPCR (**H**) and its protein levels by Western blotting (**I**). (**F**,**I**) While a representative immunoblot is shown, the intensities of immunoreactive bands from 4 independent experiments were semiquantified by densitometry and are summarized as mean ± SEM on the right. (**E**,**F**,**H**,**I**) * *p* < 0.05, † at least *p* < 0.01 vs. untreated controls (n = 4).

Since IL-18-induced TRAF3IP2 expression but suppressed RECK in ASMC (Figure 1), we next investigated potential mechanisms underlying its differential effects on TRAF3IP2 and RECK. It has been previously reported that miR-30a directly targets TRAF3IP2 [48], and miR-342 indirectly regulates RECK expression [49]. Interestingly, the results in Figure 5A (experimental design in Figure 5A) show that exposure to IL-18 inhibited miR-30a expression in ASMC (Figure 5B), and pretreatments with the p38 MAPK inhibitor SB239063 and the JNK inhibitor SP600125 each partially restored its expression (Figure 5B). However, the ERK inhibitor SCH772984 did not significantly modulate its expression. Similar to its inhibitory effects on miR-30a (Figure 5B), exposure to IL-18 also suppressed miR-342 expression in part via p38 MAPK and JNK (Figure 5C). All three inhibitors inhibited the IL-18-induced activation of respective targets in ASMC without affecting viability. These results suggest that IL-18 inhibits the induction of miR-30a and miR-342 in ASMC, in part via p38 MAPK and JNK (Figure 5).

### 3.6. miR-30a and miR-342 Mimics Reverse IL-18-Induced TRAF3IP2 Expression and RECK Suppression

Since IL-18 induced TRAF3IP2 expression (Figure 1B,C) and miR-30a suppression (Figure 5B), we next investigated whether a miR-30a mimic reverses IL-18-induced TRAF3IP2 upregulation (experimental design in Figure 5D). The results show that while IL-18 upregulated TRAF3IP2 mRNA expression (Figure 5E) and protein levels (Figure 5F) in ASMC, transfection with a miR-30a mimic markedly inhibited TRAF3IP2 upregulation at both mRNA and protein levels. Since miR-342 targets RECK expression in an indirect manner, we next investigated if a miR-342 mimic restores its expression (experimental design in Figure 5G). Consistent with the results in Figure 1, IL-18 suppressed RECK expression at both mRNA (Figure 5H) and protein levels (Figure 5I), and this effect was reversed following transfection with a miR-342 mimic. These results indicate that IL-18 regulates TRAF3IP2 and RECK expression in part via miR-30a and miR-342, respectively, in ASMCs (Figure 5).

### 3.7. EF24, a Curcumin Analog, Inhibits IL-18-Induced ASMC Proliferation and Migration without Affecting Cell Viability

**Figure 6:**
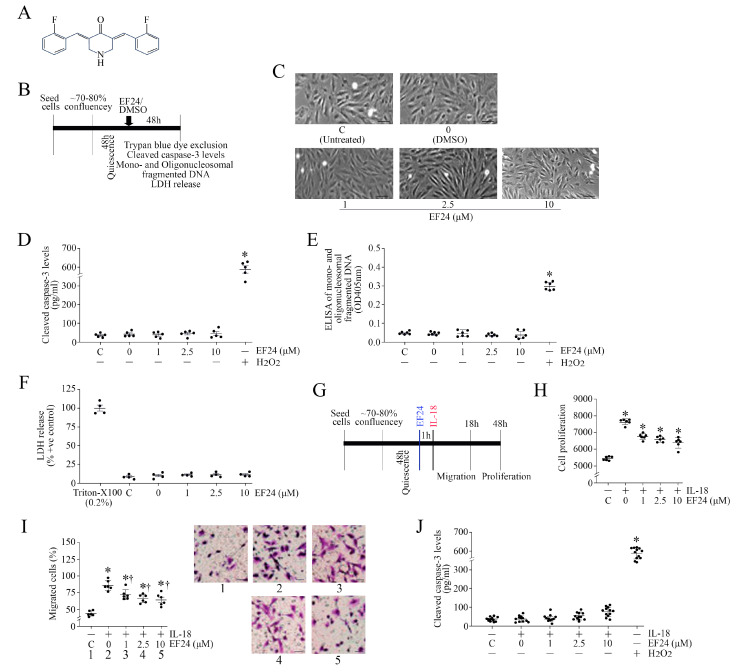
EF24 inhibits IL-18-induced ASMC proliferation and migration, without affecting cell viability. (**A**) Chemical structure of EF24, a curcumin analog. (**B**–**F**) EF24 is not cytotoxic to ASMC at the concentrations used. Quiescent ASMCs were exposed to EF24 at the indicated concentrations for 48 h (experimental design in (**B**)). Cell viability was analyzed by trypan blue dye exclusion (**C**), cleaved caspase-3 levels by ELISA (**D**) with H_2_O_2_ (100 μM) serving as a positive control, ELISA of mono-oligonucleosomal fragmented DNA (**E**) with H_2_O_2_ (100 μM) serving as a positive control, and LDH release with LDH release in response to 0.2% Triton-X100 being considered as 100% (**F**). DMSO (0.1%) alone served as a solvent control (depicted as “0”). Cells without any treatment served as a control (**C**). (**D**–**F**) * *p* < 0.01 vs. untreated controls or treated with DMSO alone. (**G**–**H**) EF24 inhibits IL-18-induced ASMC proliferation and migration. Quiescent ASMCs were incubated with EF24 at various concentrations ranging from 1 to 10 μM in DMSO for 1 h, followed by the addition of IL-18 at 10 ng/mL for 48 h (experimental design in (**G**)). Cell proliferation was analyzed by the CyQUANT Cell proliferation assay (**H**). Cell migration was analyzed by Boyden chamber assay after 18 h ((**I**) The insets show representative images of Matrigel™ transwell invasion). The combination of IL-18 and EF24 did not affect cell viability (**J**). (**C**,**I**) Scale bars, 20 μM. (**H**,**I**) * *p* < at least 0.05 vs. untreated controls, † *p* < 0.05 vs. IL-18 without EF24 (n = 6). (**J**) * *p* < 0.01 vs. untreated controls (n = 12).

Curcumin exerts multiple biological effects, including antioxidant, anti-inflammatory, and pro-apoptotic effects, in a cell type-specific manner [26,27,28,29,30,31,32]. However, its oral absorption and bioavailability are poor. Therefore, several analogs have been developed, including EF24 (Figure 6A), with improved bioavailability and bioactivity profiles [32,33,34,35,36]. However, EF24 has been shown to promote cell death in various cancer cells. It is not known whether EF24 also affects ASMC viability (experimental design in Figure 6B). Therefore, we determined the effects of EF24 on ASMC viability using four different methods: (1) trypan blue dye exclusion, (2) cleaved caspase-3 levels with 100 μM H_2_O_2_ serving as a positive control, (3) Cell Death Detection ELISA^PLUS^ that quantifies mono- and oligonucleosomal fragmented DNA with 100 μM H_2_O_2_ serving as a positive control, and (4) LDH release into culture media, where the release of LDH in response to 0.2% Triton-X100 is considered as 100%. All four methods demonstrated that, unlike the positive controls used, exposure to EF-24 at the indicated concentrations of 1, 2.5 and 10 μM for 48 h did not affect cell viability (Figure 6C–F). We then investigated if exposure to EF24 inhibits IL-18-mediated ASMC proliferation and migration (experimental design in Figure 6G). ASMCs were pretreated with EF24 at the indicated concentrations for 1 h, followed by the addition of IL-18 at 10 ng/mL (experimental design in Figure 6B). Confirming our earlier results, IL-18 significantly induced ASMC proliferation (Figure 6H) and migration (Figure 6I, the inset shows representative images of Matrigel™ Transwell invasion assay), and these effects were markedly attenuated by EF24 in a dose-dependent manner, with a marked suppression observed at a 2.5 μM concentration. Increasing its concentration to 10 μM did not further suppress ASMC proliferation and migration. Importantly, the reduced proliferation and migration following EF24 treatment were not due to reduced cell viability, as evidenced by the low levels of cleaved caspase-3 (Figure 6J). However, H_2_O_2_ used as a positive control significantly increased cleaved caspase-3 levels (Figure 6J). These results indicate that EF24 inhibits IL-18-mediated ASMC proliferation and migration without affecting cell viability (Figure 6).

### 3.8. EF24 Inhibits IL-18-Induced TRAF3IP2 Expression and RECK Suppression

**Figure 7:**
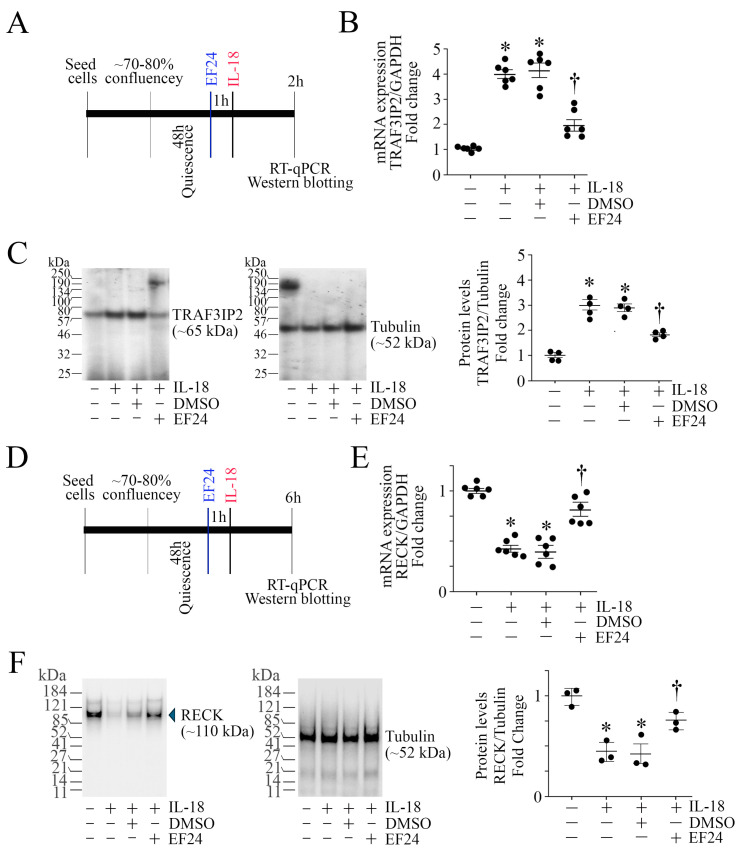
EF24 reverses IL-18-induced upregulation in TRAF3IP2 expression and RECK suppression. (**A**–**C**) EF24 blunts IL-18-induced TRAF3IP2 expression. Quiescent ASMCs were treated with EF24 (2.5 μM in DMSO for 1 h) prior to IL-18 addition at 10 ng/mL for 3 h (experimental design in (**A**)). DMSO alone (0.025%) served as a solvent control. TRAF3IP2 mRNA expression was analyzed by RT-qPCR (**B**) and its protein levels by Western blotting (**C**). (**D**–**F**) EF24 restores IL-18-induced RECK suppression. Quiescent ASMCs were treated with EF24 (2.5 μM in DMSO for 1 h) prior to IL-18 addition at 10 ng/mL for 6 h (experimental design in (**D**)). RECK mRNA expression was analyzed by RT-qPCR (**E**) and its protein levels by Western blotting (**F**). (**C**,**F**) While a representative immunoblot is shown, the intensities of immunoreactive bands from 3–4 independent experiments were semiquantified by densitometry and are summarized as mean ± SEM on the right. (**B**,**E**) * *p* < at least 0.01 vs. Untreated; † *p* < at least 0.05 vs. IL-18 or IL-18+DMSO (n = 3–4), (**C**,**F**) * *p* < 0.05 vs. Untreated; † *p* < 0.05 vs. IL-18 or IL-18+DMSO.

Our results show that IL-18 induced ASMC proliferation, migration, and proinflammatory phenotype changes in part via TRAF3IP2 induction and RECK suppression (Figure 3 and Figure 4, respectively). Therefore, we sought to determine whether EF24 treatment reverses these effects (experimental design in Figure 7A). Indeed, our results show that pretreatment with EF24 at 2.5 μM significantly suppressed IL-18-mediated TRAF3IP2 mRNA expression (Figure 7B), as well as its protein levels (Figure 7C). Further, pretreatment with EF24 (experimental design in Figure 7D) reversed IL-18-induced RECK suppression at both mRNA (Figure 7E) and protein levels (Figure 7F). These results indicate that pretreatment with EF24 inhibits IL-18-induced TRAF3IP2 upregulation and RECK suppression in ASMC (Figure 7).

### 3.9. EF24 Inhibits IL-18-Induced Proinflammatory Phenotype Changes in ASMC

**Figure 8:**
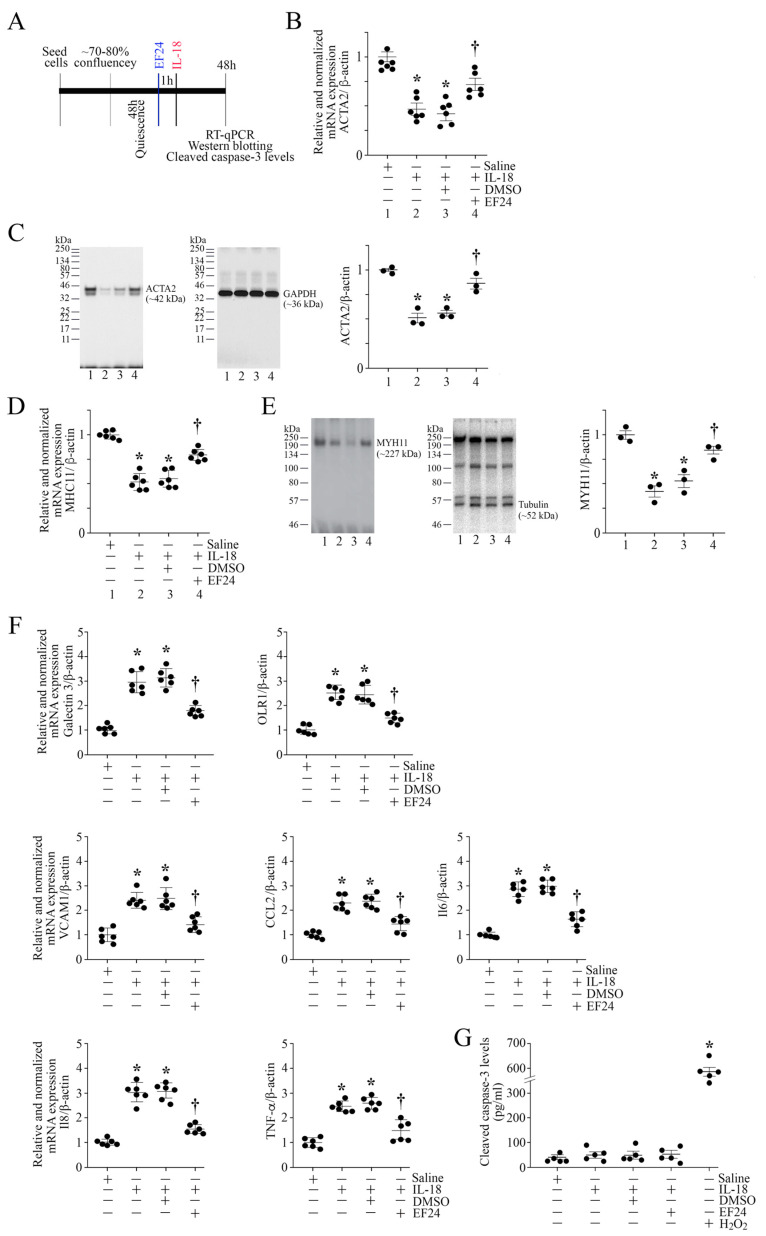
EF24 reverses ASMC proinflammatory phenotype without affecting cell viability. (**A**–**F**) Pretreatment with EF24 restores the IL-18-induced suppression of SMC markers and inhibits the expression of proinflammatory phenotype markers, without significantly modulating cell viability. Quiescent ASMCs were treated with EF24 (2.5 μM for 1 h in DMSO) prior to the addition of IL-18 at 10 ng/mL for 48 h (experimental design in (**A**)). The expression levels of SMC markers ACTA2 ((**B**) mRNA, (**C**) protein levels) and MYH11 ((**D**) mRNA, (**E**) protein levels) were analyzed by RT-qPCR and Western blotting, and those of proinflammatory phenotype markers Galectin 3, Olr1, VCAM, CCL2, IL-6, IL-8, and TNF-α (**F**) were analyzed by RT-qPCR using TaqMan™ probes. Cell viability was assessed by analyzing cleaved caspase-3 levels using a Caspase-3 (Cleaved) Human ELISA kit (**G**). H_2_O_2_ (100 μM) for 24 h served as a positive control and induced a significant increase in cleaved capase-3 levels. (**C**,**E**) While a representative immunoblot is shown, the intensities of immunoreactive bands from three independent experiments were semiquantified by densitometry and are summarized on the right. (**B**,**D**,**F**,**G**) * *p* < at least 0.01 vs. Untreated; † *p* < at least 0.05 vs. IL-18 or IL-18+DMSO (n = 5–6), (**C**–**E**) * *p* < 0.05 vs. Untreated; † *p* < 0.05 vs. IL-18 or IL-18+DMSO (n = 3).

The data in Figure 3 show that exposure to IL-18 promotes the loss of SMC markers and the induction of proinflammatory phenotype changes in ASMC. Since EF24 blunted IL-18’s pro-mitogenic and pro-migratory effects (Figure 7), we next investigated whether EF24 also inhibits the proinflammatory phenotype changes in ASMCs (experimental design in Figure 8A). Confirming earlier results (Figure 3), IL-18 inhibited mRNA expression and protein levels of SMC markers ACTA2 (Figure 8B,C) and MYH11 (Figure 8D,E), and induced the proinflammatory markers Galectin-3, OLR1, VCAM1, CCL2, IL-6, IL-8, and TNF-αin ASMC (Figure 8F), and pretreatment with EF24 restored the expression levels of the SMC markers ACTA2 and MYH11 and reversed the proinflammatory marker gene expression. Since DMSO is used as a solvent, and DMSO by itself has been shown to exert anti-inflammatory effects, we also determined whether DMSO alone inhibits the expression levels of the proinflammatory marker CCL2 in ASMCs. The results in Supplementary Figure S2 show that fresh DMSO at the indicated final concentrations did not affect basal CCL2 expression. Also, DMSO at 0.1% did not significantly modulate IL-18-induced CCL2 expression, indicating that the inhibitory effects of EF24 on migration and proliferation were due to EF24, and not due its solvent control DMSO. Together, these results indicate that EF24 blunts the IL-18-induced proinflammatory phenotype switching of ASMC (Figure 8).

### 3.10. EF24 Inhibits MMP Expression in ASMC

**Figure 9:**
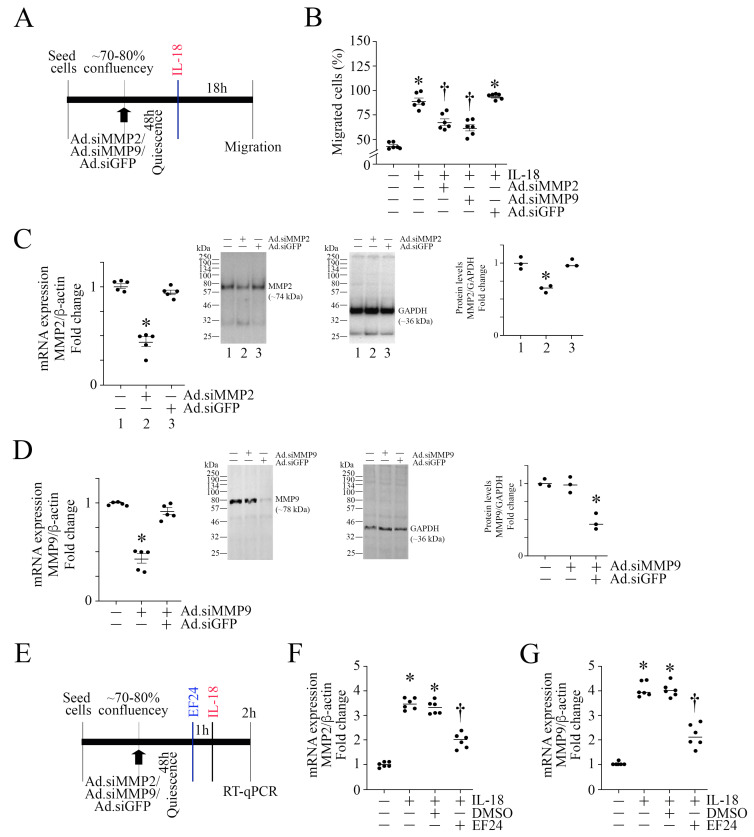
EF24 inhibits IL-18-induced MMP2 and MMP9 expressions. (**A**–**D**) Silencing MMPs 2 and 9 inhibits IL-18-induced ASMC migration. ASMCs were transduced with adenoviral vector-expressing human MMP2 or MMP9 shRNA (moi10 for 48 h), made quiescent in basal medium containing ITS-G 1X supplement and then treated with IL-18 at 10 ng/mL for 18 h (experimental design in (**A**)). Ad.siGFP at moi 10 for 48 h served as a control. Cell migration was analyzed by the Boyden chamber assay (**B**). (**C**,**D**) Knockdown of MMPs 2 and 9 was analyzed by RT-qPCR and their protein levels by Western blotting (insets). (**C**,**D**) While a representative immunoblot is shown, the intensities of immunoreactive bands from 3 independent experiments were semiquantified by densitometry and are summarized as mean ± SEM on the right. (**E**–**G**) EF24 inhibits IL-18-induced MMP expression. SMCs made quiescent in basal medium containing ITS-G 1X supplement for 48 h were incubated with EF24 (2.5 μM) for 1 h followed by IL-18 at 10 ng/mL for 2 h (experimental design in (**E**)). MMP2 (**F**) and MMP9 (**G**) mRNA expressions were analyzed by RT-qPCR. (**B**,**F**,**G**) * *p* < at least 0.01 vs. Untreated; † *p* < at least 0.05 vs. IL-18 or IL-18+siGFP (n = 3–6); ((**C**,**D**) left) * *p* < 0.001 vs. Untreated; ((**C**,**D**) right) * *p* < 0.05 vs. Untreated.

MMPs play a critical role in ECM proteolysis and SMC migration. We have previously demonstrated that IL-18 stimulates human coronary artery and saphenous vein SMC via the induction of MMP9 [39,50]. Both gelatinases MMP2 and MMP9 are shown to play a role in cell migration. Since EF24 inhibited IL-18-induced ASMC migration (Figure 6I), we next investigated whether EF24 inhibits ASMC migration by targeting MMPs 2 and 9 (experimental design in Figure 9A). Confirming our earlier results (Figure 2C), IL-18 induced ASMC migration, an effect markedly attenuated by MMP2 and MMP9 knockdown (Figure 9B; knockdown of MMPs 2 and 9 are confirmed by RT-qPCR and Western blotting as shown in Figure 9C and Figure 9D, respectively). Importantly, EF24 (experimental design in Figure 9E) at 2.5 μM concentration significantly inhibited IL-18-mediated MMP2 (Figure 9F) and MMP9 (Figure 9G) mRNA expression in ASMCs, indicating that EF24 inhibits ASMC migration in part by inhibiting MMPs 2 and 9 expression (Figure 9).

## 4. Discussion

The proinflammatory cytokine IL-18 has been implicated in the pathophysiology of atherosclerosis in preclinical models [16,51]. In fact, *Il18* gene deletion [14] and the forced expression of the IL-18 binding protein, a natural inhibitor of IL-18 [16], both decreased atherosclerosis in apolipoprotein E-deficient mice. In contrast, the administration of IL-18 accelerated atherosclerosis in apolipoprotein E-deficient mice via an interferon-γ (IFNγ)-dependent mechanism [52], suggesting that likely targets of IL-18 during atherogenesis are immune cells, such as CD4+ T cells [14,53], eliciting IFNγ production and the subsequent upregulation of pro-inflammatory responses [54]. Of note, IL-18 has also been suggested to play a role in plaque instability [16]. Interestingly, IL-18 and its receptor (IL-18 receptor/IL-18R) are expressed in other cell types within an atherosclerotic plaque, including endothelial cells, macrophages, and SMCs [16], suggesting pleiotropic proinflammatory effects of IL-18 in a wider variety of cells. Considering the integral role of SMCs in providing structural support to atheroma, we sought to investigate IL-18’s potential effects on ASMCs, which can be detrimental, inducing instability in plaques and subsequent life-threatening clinical events.

**Figure 10:**
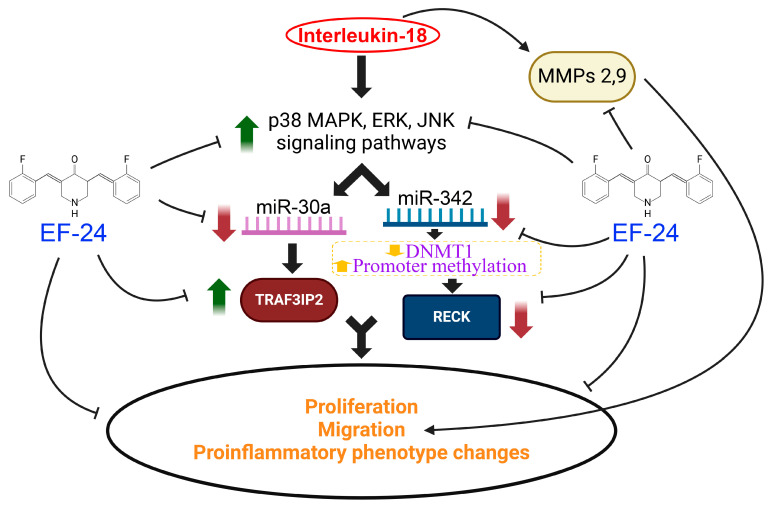
Schematic showing that EF24, a curcumin analog with better bioavailability and biologic activity, inhibits the proinflammatory IL-18-induced primary human aortic smooth muscle cells’ (ASMCs) proliferation, migration, and proinflammatory phenotype changes. EF24 inhibits the stress-activated kinase-dependent miR-30a and miR-342 inhibition, TRAF3IP2 upregulation and RECK suppression. While miR-30a mimic reverses IL-18-induced TRAF3IP2 upregulation, the miR-342 mimic restores IL-18-mediated RECK suppression, potentially via reduced DNMT1 expression and promoter demethylation (dashed purple box). While IL-18 promotes ASMC migration and proliferation, these effects were reversed by TRAF3IP2 knockdown or the ectopic expression of RECK. Further, while IL-18 induced ASMC migration in part via induction of the gelatinases MMP2 and MMP9, these effects were inhibited by EF24. Moreover, EF24 restores IL-18-mediated suppression in SMC markers and blunts the expression of the proinflammatory phenotype markers. These results suggest that the curcumin analog EF24 reverses IL-18-induced ASMC proliferation and migration and proinflammatory phenotypic changes by targeting TRAF3IP2 and restoring RECK expression. These results suggest the therapeutic potential of EF24 in vascular inflammatory and proliferative diseases, including atherosclerosis.

Our results show that exposure to IL-18 promotes ASMC proliferation and migration, along with TRAF3IP2 upregulation and RECK downregulation. Importantly, the shRNA-mediated TRAF3IP2 knockdown and forced expression of RECK by adenoviral gene transduction each reversed IL-18’s effects, suggesting that TRAF3IP2 and RECK are critical regulators of IL-18’s effects in ASMCs. Intriguingly, IL-18 upregulated a set of genes that are collectively thought to serve as markers of pro-inflammatory cell phenotype (CCL2, IL-6, VCAM-1, Galectin3, TNF-α and OLR1; Figure 4), while downregulating SMC marker genes (ACTA2, MYH11; Figure 4), suggesting that IL-18 promotes the phenotypic modulation of ASMC to an atherosclerosis-promoting inflammatory phenotype. These results indicate that IL-18 induces the migration, proliferation and pro-inflammatory phenotype-switching of SMCs in a plaque, and therefore is pro-atherogenic (Figure 10). Though we have not investigated the molecular mechanisms underlying the IL-18-induced upregulation in the gene expression of proinflammatory markers CCL2, IL6, IL8 and TNF-α in ASMC, we hypothesize that the activation of ubiquitously expressed redox-sensitive transcription factors like NF-κB and AP-1 could contribute to their induction, as we have shown previously in various cell types, including primary cardiac fibroblasts [55], primary cardiomyocytes [56], cardiac endothelial cells [57], and human coronary artery SMC [58].

Our results also indicate that while TRAF3IP2 is a positive regulator, RECK serves as a negative regulator of IL-18’s atheropromotive effects, and interventions that suppress TRAF3IP2 expression or sustain RECK levels could reverse IL-18’s proinflammatory and proatherogenic effects. Therefore, TRAF3IP2 and RECK could serve as effective therapeutic targets in atherosclerosis development and progression. Intriguingly, IL-18-mediated TRAF3IP2 induction and RECK suppression are mediated through the downregulation of miR-30a and miR-342, respectively. Further investigations have revealed that IL-18 inhibited both miRNAs in part via the activation of stress-activated protein kinases p38 MAPK and JNK. Of note, Wang et al. previously reported that the expression of miR-342 is downregulated in tissues and cell lines of human colon cancer [49]. They further demonstrated that restoring miR-342 expression inhibited DNMT1 expression by directly targeting its 3′ untranslated region, resulting in RECK reactivation by promoter demethylation.

Curcumin has been recognized for its beneficial biological and pharmacological effects both in vivo and in vitro, which include antioxidant [26,59], anti-inflammatory [60], antimicrobial [61], and anti-cancer [62,63] effects. Though several clinical trials have been completed or are ongoing to determine its bioavailability and effects in combination with other drugs in various human diseases (https://clinicaltrials.gov/search?term=Curcumin, accessed on 20 September 2024), its therapeutic potential is limited due its low bioavailability, resulting from poor gastrointestinal absorption [64,65] and short half-life in circulation [66]. In contrast, EF24, a synthetic monocarbonyl analogue of curcumin [67], exhibits efficient gastrointestinal absorption and extensive tissue distribution along with efficient metabolism, thus ensuring improved bioavailability and bioactivity [68]. Here, our results show that EF24 inhibited IL-18-induced ASMC proliferation and migration without compromising cell viability. Taking it into account that the serum curcumin levels can reach the micromolar range (2.30 ± 0.26 μg/mL;~6.2 μM) for a single oral dose of 10 g [69], the experimental dose of EF24 (0–10 μM) is within a physiological range. Therefore, our results suggest a therapeutic potential of EF24 in vascular proliferative and inflammatory diseases, where fibrotic and inflammatory responses by SMCs are the underlying mechanisms, such as atherosclerosis. In fact, anti-inflammatory effects of curcumin have been reported previously [70]. It was shown that curcumin can suppress NF-κB activation [71], which may lead to the downregulation of COX-2 and iNOS [71,72].

Curcumin is also reported to regulate AP-1 activation [73,74]. AP-1 is well known to play a role in the induction of inflammatory mediators [75,76]. Our results of EF24 attenuating inflammatory cytokine gene expression (Figure 8) are consistent with EF24’s inhibitory effects on NF-κB and/or AP-1-dependent signaling pathways, while upregulating SMC marker gene expression. This raises the possibility that the administration of EF24 could preserve/reverse SMC’s differentiation status as mature SMC (i.e., contractile phenotype), opposing IL-18-induced proinflammatory phenotype modulation. Our future studies will, however, investigate potential mechanisms by which EF24 reverses the SMCs’ proinflammatory phenotype to a contractile phenotype.

Our results also demonstrate that IL-18 upregulates TRAF3IP2 expression, an essential signaling mediator of fibrotic responses from cardiac fibroblasts and SMCs [20,39]. IL-18 upregulates TRAF3IP2 expression in part via the activation of MAPK-dependent signaling pathways (p38 and JNK). Our novel finding here indicates that IL-18-mediated miR-30a downregulation is an underlying mechanism of TRAF3IP2 upregulation, and targeting p38 or JNK reversed its inhibitory effects, while a miR-30a mimic downregulated TRAF3IP2 expression. These results contrast with a previous report, which demonstrated that miR-30a directly targets TRAF3IP2 and blunts IL-17-mediated inflammatory cytokine and chemokine expression in HeLa cells, an immortalized human cervical cancer cell line [48], suggesting that its effects may be cell type-specific. Moreover, it is largely unknown how miR-30a expression is regulated; it has been shown that the Yes-Associated Protein (YAP), which is a downstream transcription regulator of the Hippo pathway, positively regulates the transcription of miR-30a [77]. Of note, the Hippo/YAP pathway has been shown to play a role in atherosclerosis [49]. Our finding of the MAPK-dependent regulation of miR-30a is novel, and further investigations are warranted to better understand its expression and regulation in ASMCs.

In addition to inhibiting miR-30a expression, IL-18 also downregulated miR-342 expression in ASMC, and a miR-342 mimic reversed RECK suppression. Of note, miRNA-342 has been shown to be downregulated in colorectal cancer tissues and cell lines, and restoring its expression while increasing RECK expression markedly reduced the proliferation and migration of a colorectal cancer cell line [49]. Interestingly, the expression of miR-342 has also been shown to be downregulated in the peripheral blood mononuclear cells of type 1 diabetic patients and inversely correlated with pro-inflammatory cytokine expression, including IL-6, IL-8, and TNF-α [78], suggesting its anti-inflammatory and potentially anti-atherogenic role. However, its effects appear to be cell type-specific. For example, exposure to hydrogen peroxide upregulated miR-342 expression in cultured vascular endothelial cells, and the forced expression of an miR-342 mimic promoted endothelial cell apoptosis. Our finding of miR-342-mediated RECK regulation is in line with the anti-inflammatory role of RECK [79], in addition to its anti-proliferative and anti-migratory effects. More recently, it has been reported that exosome-derived miR-342 from intermittent hypoxia-exposed valvular interstitial cells inhibits the eIF2α/ATF4 signaling pathway and the osteogenic differentiation and progression of calcified aortic valve disease (CAVD) [80], an inflammatory disease with similarities to atherosclerosis. Since miR-342 is a positive regulator of RECK expression [49], it is plausible that RECK could play a protective role in CAVD.

Our study has some limitations. The current study was conducted in primary human aortic SMC, in which IL-18 elicited proinflammatory, pro-mitogenic, and pro-migratory effects, and promoted proinflammatory phenotype switching. These studies, which may not recapitulate the complex biology involved in atherosclerosis development and progression in vivo, nevertheless demonstrate that exposure to the curcumin derivative EF24 exerts promising anti-atherosclerotic effects by reversing some of the above deleterious effects of IL-18 in this cell type. Since EF24 has superior bioavailability and biological activity, future studies will aim to investigate its potential as an anti-atherosclerotic agent in preclinical models. A second limitation is that we exposed ASMC to EF24 prior to IL-18 addition (pretreatment). It is not, however, known whether EF24 is equally effective when added after IL-18. We will conduct these studies in future. Another potential limitation of our study is that though we have demonstrated that IL-18-mediated suppression in miR-30a and miR-342 expression results in TRAF3IP2 upregulation and RECK suppression, respectively, we did not fully investigate the underlying molecular mechanisms. We did, however, demonstrate that the stress-activated kinases p38 MAPK and JNK played a role in the IL-18-mediated suppression of these two miRs. Further mechanistic studies are needed to better understand how these two miRs are regulated by IL-18, how these miRs contribute to TRAF3IP2 induction and RECK suppression, and how EF24 interacts with these pathways.

## 5. Conclusions

Smooth muscle cell migration, proliferation and proinflammatory phenotype modulation contribute to vascular proliferative diseases, including atherosclerosis. IL-18 is a proinflammatory pleiotropic cytokine, whose increased expression has been shown to play a role in the development and progression of atherosclerosis. Here, we show that exposure to IL-18 promotes the proliferation, migration, and proinflammatory phenotype switching of ASMCs (Figure 10), all of which contribute to atherosclerosis development. IL-18 exerted these biological effects in part via upregulation of the pro-inflammatory molecule TRAF3IP2 and downregulation of RECK, an anti-inflammatory and anti-fibrotic molecule. Our results also show that EF24, a curcumin analog, reversed the atheropromotive effects of IL-18 by reversing TRAF3IP2 induction and RECK suppression through p38 MAPK and JNK-dependent miR-30a and miR-342 regulation. Our results suggest that EF24, either alone or as an adjunctive therapy, has the potential to delay the development and progression of atherosclerosis (Figure 10).

### Future Directions

Recently, several derivatives of EF24 with better bioavailability have been reported [33,34,35,36]. Therefore, our future studies will investigate whether these newer derivatives of EF24 are more potent that EF24 itself in exerting anti-inflammatory and anti-atherosclerotic effects both in vitro and in vivo.

## Data Availability

Raw data are available from the corresponding author upon request.

## Supplementary Materials

The following supporting information can be downloaded at: https://www.mdpi.com/article/10.3390/cells13201673/s1, Figure S1: Transfection with adenoviral vectors, miRNA mimics and their negative control (NC) at the indicated doses and duration did not affect cell shape or viability or adherence as determined by trypan blue dye exclusion; Figure S2: DMSO up to 0.1% did not significantly affect basal CCL2 mRNA expression. DMSO at 0.1% also did not significant affect IL-18-induced CCL2 expression.

## Abbreviations

ACTA2, Actin Alpha 2, Smooth Muscle; AP-1, Activator Protein-1; CCL2, C-C Motif Chemokine Ligand 2; COX-2, Cyclooxygenenase-2; DMSO, dimethylsulfoxide; DNMT1, DNA Methyltransferase 1; ERKi, Extracellular Signal-Regulated Kinase (ERK) inhibitor; EF24, ((3E,5E)-3,5-bis[2-fluorophenyl (methylene]-4piperidinone); GAPDH, Glyceraldehyde-3-Phosphate Dehydrogenase; SMC, Smooth Muscle Cells; ASMC, aortic SMC; JNKi, c-Jun N-terminal Kinase inhibitor; p38 MAPKi, p38 Mitogen-Activated Protein Kinase inhibitor; IL, Interleukin; iNOS, inducible Nitric Oxide Synthase; rhIL-18, Recombinant human Interleukin-18; LDH, lactose dehydrogenase; miR, micro RNA; p-ERK, phospho-ERK; MMP, Matrix Metallopeptidase or Matrix Metalloproteinase; MYH11, Myosin Heavy Chain 11; NF-κB, Nuclear Factor kappa-light-chain-enhancer of activated B cells; Olr1, Oxidized Low-Density Lipoprotein Receptor 1; TNF, Tumor Necrosis Factor; TRAF3IP2, TRAF3 Interacting Protein 2; TRAF, TNF Receptor Associated Factor; VCAM1, Vascular Cell Adhesion Molecule-1; kDa, Kilodaltons; U6, U6 small nuclear RNA; NC, Negative Control miRNA; RECK, REversion inducing Cysteine rich protein with Kazal motifs; siRNA, small interfering RNA; shRNA, Short hairpin RNA; Ad.GFP, adenovirus-expressing Green Fluorescent Protein; Ad.eGFP, adenovirus-expressing enhanced GFP; ITS, Insulin–Transferrin–Selenium; LGALS3, Lectin, Galactoside-Binding, Soluble.
