# Expression of Concern: Higashi et al. EF24, a Curcumin Analog, Reverses Interleukin-18-Induced miR-30a or miR-342-Dependent TRAF3IP2 Expression, RECK Suppression, and the Proinflammatory Phenotype of Human Aortic Smooth Muscle Cells. *Cells* 2024, *13*, 1673

**DOI:** 10.3390/cells15222057

**Published:** 2026-11-12

**Authors:** Cells Editorial Office

**Affiliations:** MDPI AG, Grosspeteranlage 5, 4052 Basel, Switzerland; cells@mdpi.com

With this notice, the *Cells* Editorial Office alerts readers to concerns regarding this article [1].

Following publication, concerns were raised regarding the integrity of Figure 7C. In accordance with the journal’s standard procedures, the authors were contacted and asked to provide clarification and supporting documentation.

Based on the information available, the Editorial Board concluded that the concerns relating to Figure 7C could not be fully resolved. While the authors cooperated with the investigation, they were unable to provide sufficient supporting material to fully validate the figure. The Editorial Board further determined that these concerns do not substantially affect the overall findings and conclusions presented in the article.

This Expression of Concern is issued to inform readers of the unresolved concerns regarding Figure 7C, which should therefore be interpreted with appropriate caution. Based on the information currently available, no further editorial action is warranted at this time. Should additional information become available, however, the Editorial Office and Editorial Board may reconsider the matter and take further action in accordance with MDPI’s policies.
