# Supplementary material for: EF24, a Curcumin Analog, Reverses Interleukin-18-Induced miR-30a or miR-342-Dependent TRAF3IP2 Expression, RECK Suppression, and the Proinflammatory Phenotype of Human Aortic Smooth Muscle Cells
Source: Cells. 2024 Oct 10;13(20):1673. doi: 10.3390/cells13201673 (PMC11505909; doi:10.3390/cells13201673)
Supplement: Supplementary file 1 [file cells-13-01673-s001.zip › cells-3163468-supplementary.pdf]

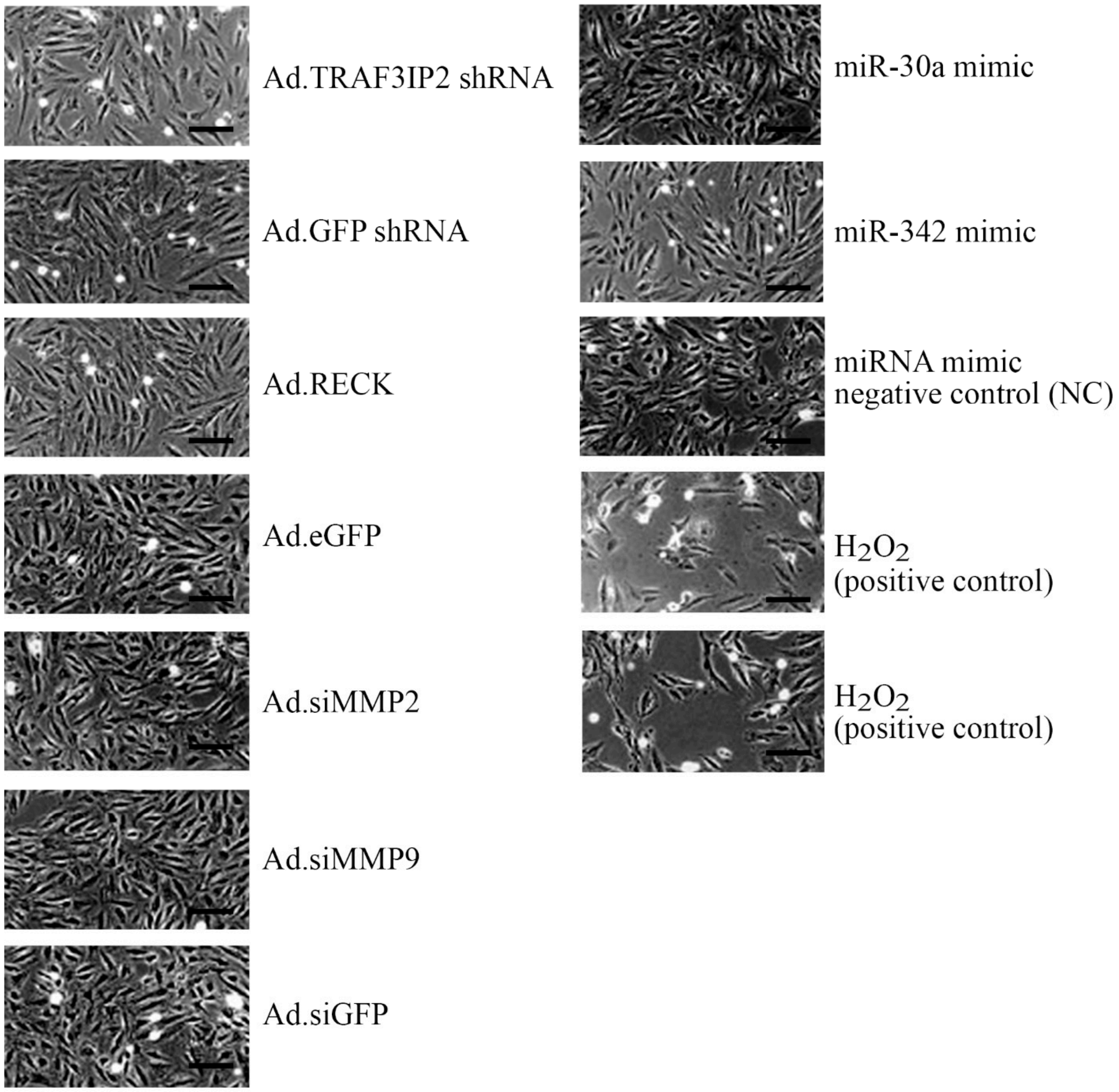

Supplementary Figure S1. Transfection with adenoviral vectors, miRNA mimics and their negative control (NC) at the indicated doses and duration did not affect cell shape or viability or adherence as determined by trypan blue dye exclusion. However, H_2O_2 (100 µM for 18 h) used as a positive control induced significant cell death, resulting in empty spaces in the last two images. Scale bar: 20 µM.

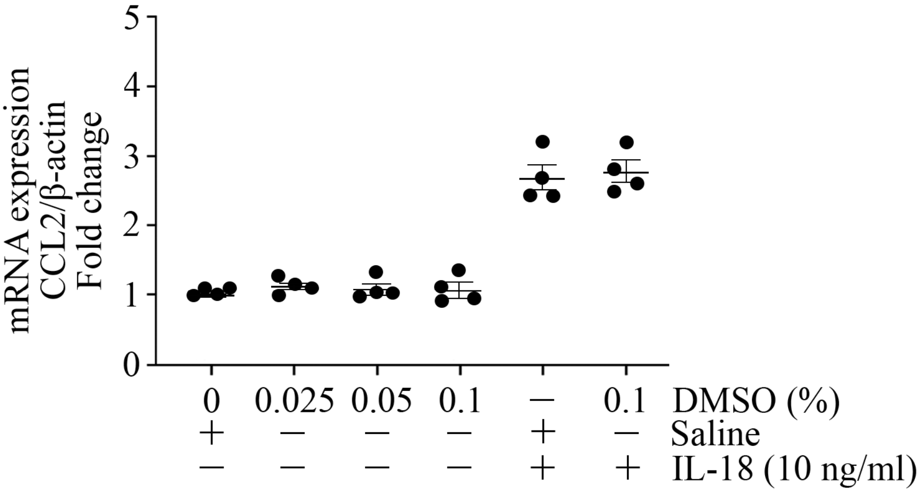

Supplementary Figure S2. DMSO was used as a solvent in experimental involving EF24 and stress-activated kinases. In experimental involving EF24, at first we performed a dose-response study, where EF24 was used between 1-10 µM concentration. Fresh DMSO was used as a solvent to dissolve EF24 and ranged between 0.025 to 0.1% based on the concentration of EF24 used.

Experimental design: Quiescent ASMC were treated with DMSO alone at a concentration thar ranged between 0.025, 0.5, and 1% for 48 h to determine if DMSO alone affects basal CCL2 expression. Experiments were also performed where quiescent ASMC were treated with 0.1% DMSO for 1 h prior to IL-18 (10 ng/ml for 48h) addition. Total RNA was isolated, converted to cDNA and analyzed for CCL2 expression by RT-qPCR using Applied Biosystems™ TaqMan® probe with β-actin serving as a loading control. The results are represented in fold change.

Results: The results show that DMSO up to 0.1% did not significantly affect basal CCL2 mRNA expression. DMSO at 0.1% also did not significant affect IL-18-induced CCL2 expression.
